# Editorial: Evolving Mechanisms of Disease Tolerance

**DOI:** 10.3389/fimmu.2019.02974

**Published:** 2019-12-20

**Authors:** Irah L. King, Maziar Divangahi

**Affiliations:** ^1^Meakins-Christie Laboratories, Department of Medicine, McGill University Health Centre, Montreal, QC, Canada; ^2^Department of Microbiology and Immunology, McGill University, Montreal, QC, Canada; ^3^McGill International TB Centre, McGill University Health Centre, Montreal, QC, Canada

**Keywords:** host defense, infection, immunity, disease tolerance, tissue damage

Within the last 150 years, morbidity and mortality due to infectious diseases has drastically decreased worldwide (1). This change in health began with the pioneering cowpox studies of Edward Jenner followed by Pasteur's germ theory and Koch's postulates that eventually led to improved hygiene strategies, the advent of vaccines and discovery of antibiotics (2). These seminal observations about the infectious origins of disease spawned the golden age of Immunology in which investigators such as Elie Metchnikoff and Paul Ehrlich broadly described the cellular and molecular mechanisms of host defense. However, it has now become clear that defense against infection extends beyond host resistance and also includes mechanisms that limit tissue damage independent of changes to pathogen burden (3). This latter strategy is referred to as disease tolerance and involves coordination between immune cells and tissue-specific structural cells to maximize host fitness in the face of disruption to homeostatic conditions (4). It is important that “disease tolerance” is not confused with the equally important concept of “immune tolerance” in which immune reactivity is inhibited by clonal deletion or silencing of antigen-specific lymphocytes (5). However, the possibility that immune tolerance and disease tolerance can operate in a complementary fashion within the same setting of infection or inflammation is certainly not excluded.

The concept of disease tolerance was introduced in 1894 by Nathan Augustus Cobb, an American plant pathologist. From his studies in wheat, he observed the ability of certain strains to yield crop despite the presence of a fungal infection or “rust.” He referred to this phenomenon as “rust-enduring” and distinguished this phenotype from “rust-resistant” wheat (6). Following Cobb's seminal observations, plant biologists rebranded the concept of endurance to disease tolerance (7). Although this concept was well-established in plant biology, it was not directly tested in mammals until more than a century later by Lars Råberg and Andrew Read. Specifically, they demonstrated that genetic variation in mice can delineate host resistance vs. disease tolerance following malaria infection (8). Soon after, molecular insights into these observations were provided by Miguel Soares' group demonstrating that tissue protection from the cytotoxic effects of malaria-induced hemolysis in mice is provided by the heme-catabolizing enzyme heme oxygenase-1 (9). In the same year, Ayres and Schneider demonstrated that simple organisms such as the fruit fly *Drosophila melanogaster* can also use disease tolerance as a host defense mechanism in the context of gram-positive and gram-negative bacterial infections (10, 11). Collectively, these studies have provided the impetus for investigating disease tolerance as an alternative and/or complementary form of host defense not only in the context of infection but also in settings of non-communicable diseases such as autoimmunity, asthma, and atherosclerosis.

This Frontiers Research Topic entitled “Evolving mechanisms of disease tolerance” aims to demonstrate how the research and our understanding of this concept is leading to, what we consider, a new golden age of infectious disease research and discovery. Considering the relevance of disease tolerance across the kingdoms of life and throughout the evolution of mammals, we have assembled exciting reviews detailing how this defense strategy is conserved from plants to humans against diverse forms of infection. Paudel and Sanfaçon return to the roots of disease tolerance by describing the mechanisms by which plants tolerate viral infection. Budischak and Cressler provide an ecological perspective on how environmental resources contribute to disease tolerance across the evolutionary spectrum. This study dovetails with the reviews of Carlos et al. whom discuss the impact of nutrient metabolism such as iron and glucose on tolerance to diverse pathogen challenge and Harbeson et al. whom examine how early-life metabolic responses impact long-term human health outcomes. Importantly, prokaryotes also utilize disease tolerance to defend against viral (i.e., bacteriophage) infection. Attention is brought to this emerging research field by Chatterjee and Duerkop highlighting the impact of bacteria-phage interactions and how they may contribute to eukaryotic immune responses.

Emphasizing the importance of disease tolerance during respiratory infection, Divangahi et al., Saelens et al., and Olive and Sassetti take aim at *Mycobacterium tuberculosis*. Their reviews emphasize how understanding the mechanisms of disease tolerance to *Mtb* infection may lead to new therapeutic strategies against Tuberculosis, the world's leading infectious killer. Beyond TB, extracellular bacterial and fungal infections of the lung remain important clinical problems, particularly in immunocompromised individuals. Disease tolerance to these pathogens are emphasized by Shourian and Qureshi in their discussion of *Cryptococcus neoformans* infection and Faure et al. whom describe mechanisms of host adaptation to *Pseudomonas aeruginosa* infection and how this response goes awry in patients with cystic fibrosis. Additionally, Allard et al. dedicate an entire article solely to describing how alveolar macrophages regulate disease tolerance in a range of settings from infection to allergy. In a complementary review, Crane et al. discuss the complex scenario in which primary respiratory viral infections can either increase or decrease disease tolerance to secondary bacterial infection depending on the immune status of the host. One important factor in the co-infection scenario are type 1 and type 2 interferons. These cytokines, reviewed by Lee and Ashkar, are rapidly and abundantly produced in response to viral and bacterial infection and have diverse roles in protective and pathogenic immune responses. In addition to highly replicative unicellular pathogens, hosts likely use distinct mechanisms of disease tolerance to protect against non-replicating, multicellular parasites. This form of pathogen challenge is the focus of two reviews in this Research Topic focusing on parasitic worm infection. Specifically, King and Li focus on the diverse mechanisms by which the mammalian host uses disease tolerance to defend as chronic intestinal helminth infection and, in a related article, Yap and Gause explore how helminths shape organ-specific strategies of disease tolerance and the consequences on heterologous infection. Finally, we have included a review by Mandl et al. in which they discuss how diverse species of bats are uniquely tolerant to viruses that are highly pathogenic to humans. Understanding the immune system of this animal will not only provide insight into mammalian mechanisms of disease tolerance, but also inform strategies that limit infection of this important pathogen reservoir. In sum, we hope that this collection will highlight recent developments related to the origins and function of disease tolerance as well as persuade the development of therapeutic strategies targeting this fundamental strategy of host defense against infectious diseases.

## Author Contributions

IK and MD contributed equally to this editorial and have approved it for publication.

### Conflict of Interest

The authors declare that the research was conducted in the absence of any commercial or financial relationships that could be construed as a potential conflict of interest.
